# Progerin, the protein responsible for the Hutchinson-Gilford progeria syndrome, increases the unrepaired DNA damages following exposure to ionizing radiation

**DOI:** 10.1186/s41021-015-0018-4

**Published:** 2015-10-01

**Authors:** Asao Noda, Shuji Mishima, Yuko Hirai, Kanya Hamasaki, Reid D. Landes, Hiroshi Mitani, Kei Haga, Tohru Kiyono, Nori Nakamura, Yoshiaki Kodama

**Affiliations:** Department of Genetics, Radiation Effects Research Foundation, 5-2 Hijiyama-Park, Minami-Ku, Hiroshima 732-0815 Japan; Department of Statistics, Radiation Effects Research Foundation, 5-2 Hijiyama-Park, Minami-Ku, Hiroshima 732-0815 Japan; Department of Integrated Biosciences, Graduate School of Sciences, The University of Tokyo, Kashiwa-no-ha 5-1-5, Kashiwa, Chiba 277-8572 Japan; Division of Virology, National Cancer Center Research Institute, 5-1-1 Tsukiji, Chuo-ku, Tokyo 104-0045 Japan; Division of Carcinogenesis and Cancer Prevention, National Cancer Center Research Institute, 5-1-1 Tsukiji, Chuo-ku, Tokyo 104-0045 Japan

**Keywords:** HGPS, Unrepairable DSB, Radiation, Cell aging, FTI

## Abstract

**Introduction:**

Progerin, the protein responsible for the Hutchinson-Gilford Progeria Syndrome (HGPS), is a partially deleted form of nuclear lamin A, and its expression has been suggested as a cause for dysfunctional nuclear membrane and premature senescence. To examine the role of nuclear envelop architecture in regulating cellular aging and DNA repair, we used ionizing radiation to increase the number of DNA double strand breaks (DSBs) in normal and HGPS cells, and analyzed possible relationship between unrepaired DSBs and cellular aging.

**Results:**

We found that HGPS cells are normal in repairing a major fraction of radiation-induced double strand breaks (M-DSBs)but abnormal to show increased amount of residual unrepaired DSBs (R-DSBs). Such unrepaired DSBs were 2.6 times (CI 95 %: 2.2–3.2) higher than that in normal cells one week after the irradiation, and 1.6 times (CI 95 %: 1.3–1.9) higher even one month after the irradiation. These damages tend to increase as the nuclear envelope become abnormal, a characteristic of both HGPS and normal human cells which undergo replicative senescence. The artificial, enforced over-expression of progerin further impaired the repair of M-DSBs, implying lamin A-associated nuclear membrane has an important role for DNA DSB repair. Introduction of telomerase gene function in HGPS cells reversed such aging phenotypes along with upregulation of lamin B1 and downregulation of progerin, which is a hallmark of young cells.

**Conclusion:**

We suggest that lamin A- or progerin-associated nuclear envelope is involved in cellular aging associated with DNA damage repair.

**Electronic supplementary material:**

The online version of this article (doi:10.1186/s41021-015-0018-4) contains supplementary material, which is available to authorized users.

## Introduction

Radiation-induced unrepairable DNA damage in the genome consists primarily of DNA double strand breaks (DSBs). In vitro, non-apoptotic normal human diploid fibroblast cells (NHDFs) bearing unrepaired DSBs undergo permanent growth arrest, but survive in this terminally differentiated state for over one year [1]. In vivo, similar conditions may occur in terminally differentiated but apoptosis-resistant cells such as muscle cells, neurons and connective tissue cells. For example, radiation-induced γH2AX foci are reported to persist in pig skin [2], mouse skin [3] and mouse pancreas [1] following exposure to high doses of radiation. This suggests that the majority of cells in tissues with very slow turnover rate may accumulate unrepaired DSBs that occur either spontaneously or following exposure to radiation; e.g., mouse pancreatic cells replicate once in about 500 days and brain or kidney cells grow even more slowly [4, 5]. Such accumulation of DSBs may cause premature aging by earlier onset of malfunction of affected tissues [1, 6]. Even under steady-state conditions, about 8 DSBs are produced per cell per day [7, 8], and a small fraction of these could potentially remain unrepaired.

It has been shown that unrepaired DSBs accumulate within nuclei and the frequency of cells with dysmorphic nuclei have been found to increase during replicative senescence [1, 9–11]. Exposure to ionizing radiation increases the frequencies of both events indicating that either the presence of unrepaired DSBs alters nuclear envelope morphology and/or function, or conversely, that a dysfunctional nuclear membrane results in increased number of unrepaired DSBs, which then causes cells to undergo permanent growth arrest.

With regards to the positive association between senescence and nuclear structure, much can be learned from studies of the premature aging syndrome, HGPS (Hutchinson-Gilford progeria syndrome). HGPS is caused primarily by a dominant point mutation of nuclear lamin A gene, which codes for a major protein of the inner nuclear mesh, in exon 11 (C to T transition at nucleotide 1824), which is a silent mutation causing Gly^608^ → Gly^608^. However, the mutated sequence creates a cryptic new splicing donor site [12–14], which results in production of a mutant lamin A protein termed “progerin”. Progerin has a 50-amino-acid internal deletion which lacks the proteolytic cleavage site necessary to remove the last 18 carboxy-terminal amino acids to generate mature lamin A.

Due to lack of the cleavage site, excision of farnesylated C-terminus from lamin A precursor protein is impaired in HGPS cells, and the protein anchors to the nuclear membrane, which in turn weakens cell growth [15, 16]. Such cells, which now lose their nuclear plasticity (nuclei of HGPS cells are known to be stiffer and more fragile than normal nuclei), become sensitive to mechanical stress [17]. Thus endothelial and smooth muscle cells in blood vessels, which are constantly exposed to high levels of mechanical and oxidative stress, may experience continuous cell damage and cell loss by necrosis, followed by compensatory cell divisions leading to accelerated arteriosclerosis and early vascular defects (cardiovascular disease) in individuals with HGPS [18, 19].

In this study we sought to confirm a possible link between nuclear deformation and unrepairable DSBs, on the hypothesis that HGPS has an exaggerated phenotype of accumulating DNA damages and dysfunctional nuclear envelope. We show an increase in endogenous and radiation-induced unrepairable DSBs in HGPS cells, especially in cells bearing dysmorphic nuclei. Moreover, our finding suggests that telomerase-mediated cell immortalization is a cell rejuvenating process associated with decreasing the nuclear dysmorphism and unrepairable damages.

## Materials and methods

### Cell culture

Two strains of NHDFs, MJ90 [1, 20, 21] (also known as HCA2) and IMR90 (Coriell Institute, Camden), and two HGPS fibroblasts, AG11513 and AG11498 (Coriell Institute, Camden) were used throughout the study. Standard culture containing MEM + 10 % FCS was used for cell growth. For the measurement of unrepairable DSB-foci in NHDFs, FCS concentration was reduced to 0.1 % to maintain the cell population under quiescent conditions because growing young NHDFs produce many tiny, DNA-damage independent γH2AX/53BP1 foci while in S/M phase, which hampers counting the precise number of foci [1]. hTERT introduction into AG11513 and AG11498 cells was performed through the hTERT-retrovirus vector (MSCVpuro-hTERT) infection in their early passages. Cells were X-ray irradiated with a Shimazu/Pantak irradiator (220 KV, 8 mA, Al 0.5 mm/Cu 0.3 mm filters) at a dose rate of 1 Gy/min. Farnesyl transferase inhibitor (FTI277, Sigma F9803) was added to the culture at a final concentration of 25 μM 3 days prior to irradiation. Plasmid transfections [22] (GFP-lamin A, GFP-progerin) were carried out with the standard electroporation method (1500 V, 25 μF) to achieve single-copy integration in HeLa S3 cells, and with the lipofection method (Trans IT, Mirus) to achieve transient and multicopy delivery in MJ90 cells.

### Immunostaining and Western blot

**Fig. 1:**
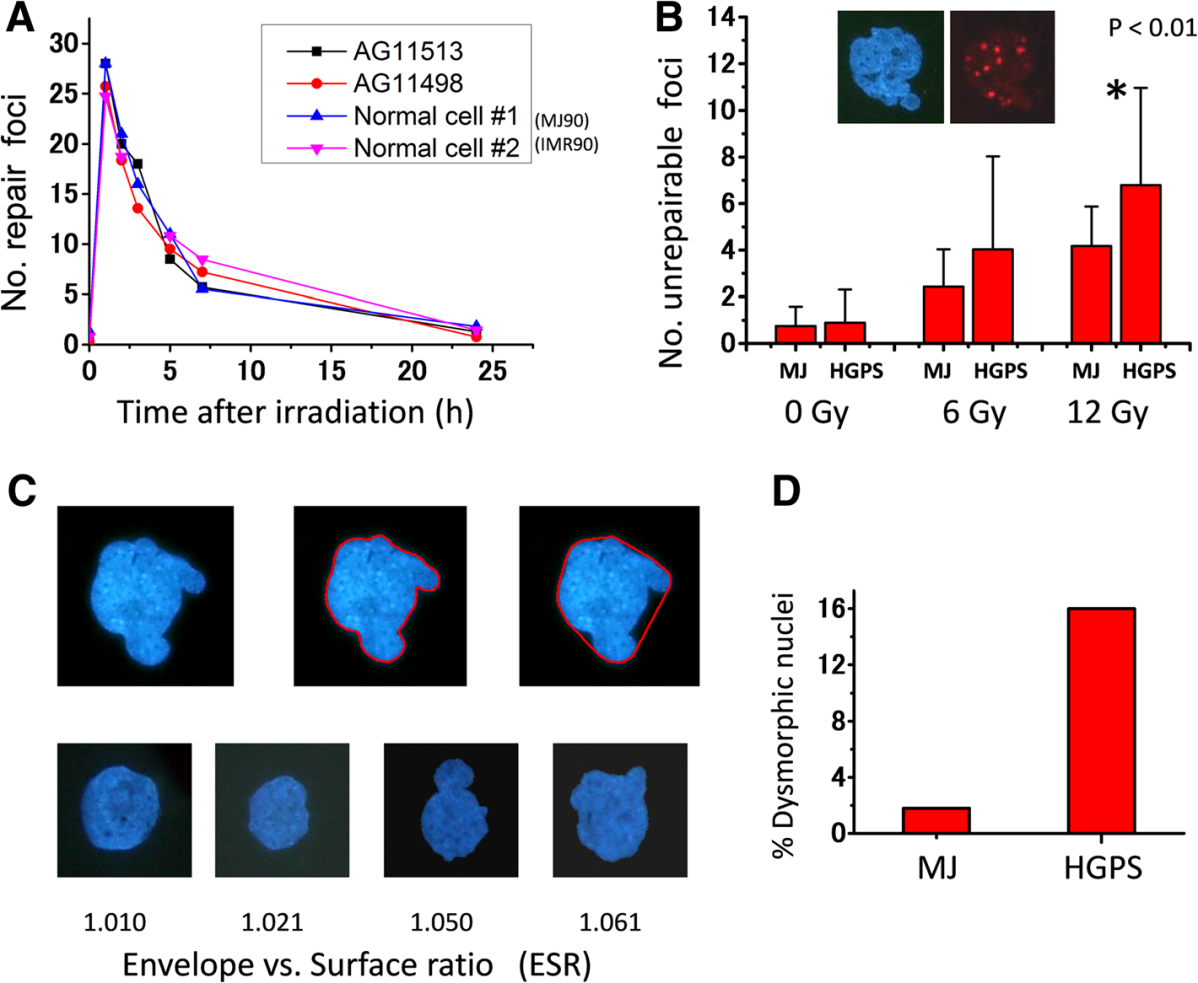
DSB repair kinetics and nuclear morphology of normal and HGPS fibroblasts. **a** 53BP1 foci formation and clearance of 1-Gy irradiated normal cells (MJ90 and IMR90) and HGPS cells (AG11513 and AG11498). **b** The number of unrepairable 53BP1 foci persisting 1 month after 6 or 12 Gy irradiation in normal (MJ90) and HGPS (AG11513) cells. Insert photos are typical deformed nucleus and its 53BP1 foci from 12 Gy-irradiated and 1 month incubated (12-Gy/1-month) HGPS cells. **c** Calculation of the degree of nuclear dysmorphology. Exact area of a nucleus shown in upper left was measured as the area surrounded by the contour line (*upper middle*), and then polygon area (*upper right*) was divided by the exact area to make the envelope vs. surface ratio (ESR). Nuclei with a ratio of 1.05 or larger were defined as dysmorphic (abnormal). **d** Percentages of dysmorphic nuclei in normal (MJ90) and HGPS (AG11513) cells. Both cell strains were at their young stages, less than 50 % of their respective in vitro replicative life span

Antibody staining was performed with standard protocols [1]. First antibodies used were, anti-LaminA/C [Mouse monoclonal clone JOL2 (ImmuQuest), Mouse monoclonal clone 4C11 (Sigma)], anti-Progerin [Mouse monoclonal clone 13A4 (Millipore)], anti-53BP1 [Rabbit polyclonal A300-272A (Bethyl)], anti-phospho-Histone H2AX [Mouse monoclonal clone JBW301 (Millipore)], anti-α-Tubulin [Mouse monoclonal cloneB-5-1-2(Sigma)]. Second antibodies were, TRITC goat anti-rabbit IgG (Jackson), Alexa 488 goat anti-mouse IgG (Invitrogen). Photo-images were taken and analyzed with the Image-Pro software 5.5 (MediaCybernetics). We prepared our own macro program for automatic detection of cell nuclei and measurements of diameter and fluorescence intensity of individual repair foci [1]. Enveloping surface ratio (ESR) (Fig. 1) was also automatically analyzed. Western analyses were performed using standard methods [1].

### Statistics

The number of unrepaired DSB foci in each cell nucleus was automatically counted as above, and mean, SD and standard t-tests were performed. To estimate the fold increase, we took the ratio of the two means of interest, and produced 95 % confidence intervals using the basic bootstrap method based on 1000 bootstrap samples [23].

## Results

### HGPS fibroblasts have impaired capability of repairing certain forms of DSBs

Liu et al. reported slower formation of 53BP1 foci following irradiation of HGPS cells whereas formation of γH2AX foci was normal [24]. We used two strains of fibroblasts derived from individuals with HGPS, AG11513 and AG11498, both carrying a typical C to T base-change mutation at nucleotide 1824 in the *lamin A* gene, and measured the number of DSB repair foci (53BP1 and γH2AX foci) at various times after irradiation with 1 Gy. Contrary to their results, our results did not indicate a repair defect in the HGPS cells with respect to formation and repair (disappearance) of 53BP1 foci (Fig. 1a). 25 to 30 53BP1 foci, which constitute the major fraction of DSBs (M-DSBs), were observed per nucleus about 1 h after irradiation. The number of foci decreased with a half-life of about 3 h and returned to close to background levels in 24 h. Similarly there was no difference in the repair of γH2AX foci between HGPS and normal cells (results not shown). The results indicate that HGPS cells are proficient at repairing common types of DSBs induced by radiation exposure. In contrast, when the numbers of unrepaired foci were examined after irradiation of cells with higher doses of X ray (6 and 12 Gy) and longer incubation time (1 month), about 1.62 times more foci were retained in HGPS versus normal cells (Fig. 1b) (*p* < 0.01, 95 % CI; 1.38 to 1.90 by boots trap test). Such foci had a larger mean size [1], similar to what we previously observed in irradiated young NHDFs. These residual unrepairable foci (R-DSBs) persisted for more than one year in normal cells [1].

### Relationship between abnormal nuclear shape and DSB repair

Dysmorphic nuclei are defined as nuclei containing bleb, hernia, invagination, and/or lobulation (Fig. 1c). Even in normal cells, dysmorphic nuclei increase in frequency during normal processes of in vitro aging and become prominent when the cells reach senescence [9, 25], (Additional file 1: Figure S1). In contrast, dysmorphic nuclei are commonly observed in HGPS cells starting from early passages of in vitro culture (Additional file 2: Figure S2), which is considered to be due to accumulation of progerin [26, 27]. Thus, in the present study, the degree of dysmorphism was quantified and its relation to the number of unrepaired 53BP1 foci was examined. The degree of nuclear dysmorphism was expressed as the ratio of nuclear area (Fig. 1c, top middle) to envelop area, defined as ESR (envelope vs. surface ratio) (Fig. 1c, top right). As shown in Fig. 1c lower panels, a nucleus with a ratio of 1.02 still looks close to normal while those ratios of 1.05 or larger look clearly abnormal. We found that about 15 % of HGPS cells were already dysmorphic (ESR ratio ≥1.05) at population doublings 20 (PDs20), which corresponds to the middle period of in vitro HGPS cell aging (Fig. 1d).

**Fig. 2:**
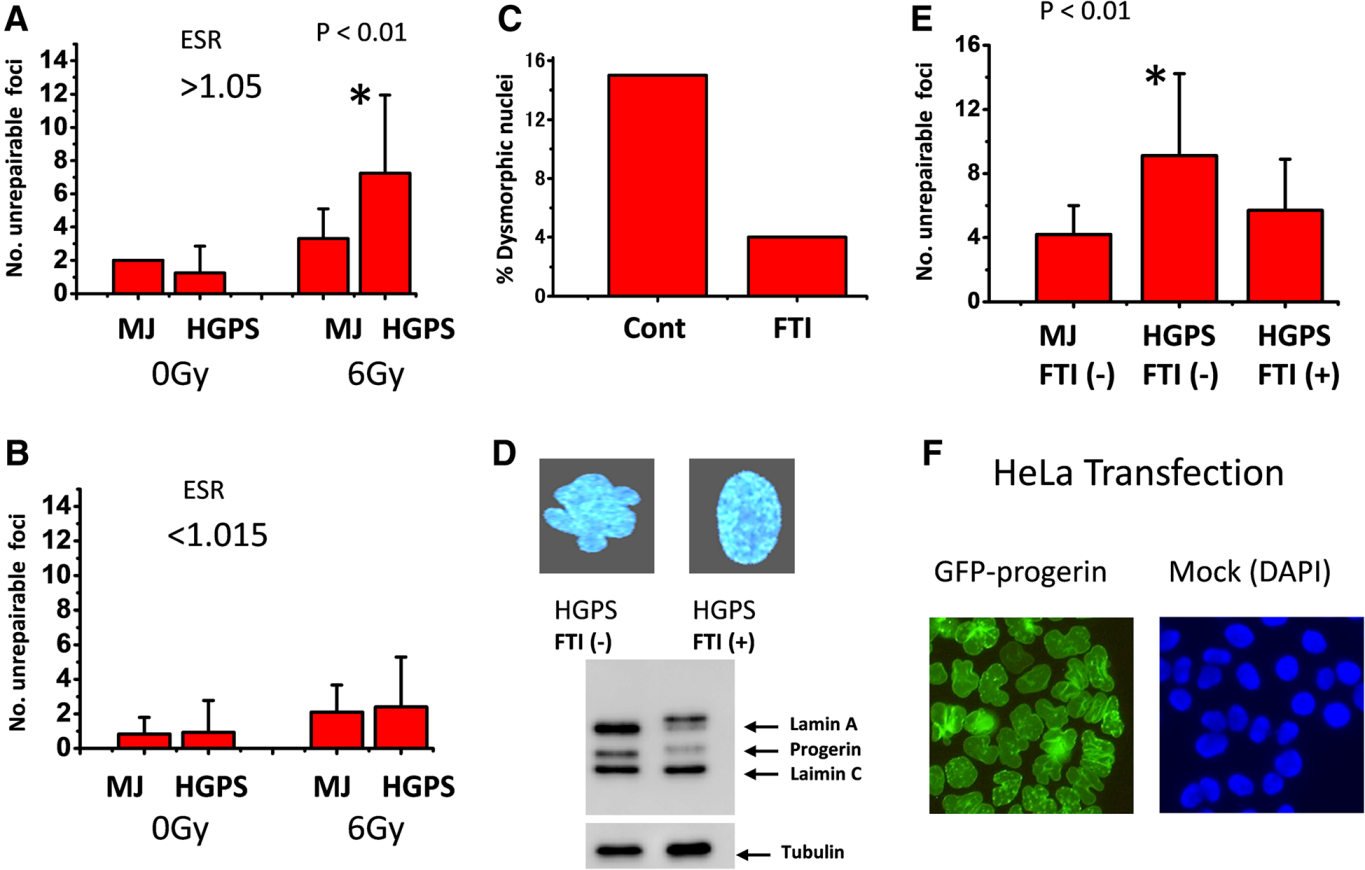
The number of unrepairable DSB foci in relation to dysmorphic nuclear shape. **a**, **b** Number of unrepairable 53BP1 foci in dysmorphic (ESR ≥1.05) and normal (ESR ≤1.015) nuclei in the 6-Gy/1-month cell population. **c** Improvement of nuclear shapes by FTI treatment (25 μM) in HGPS cells. **d** Nuclear shapes (*upper panels*) and Western blot of lamin A/C and progerin expression (*lower panels*) after the FTI treatment. The band which appeared above the lamin A band indicates unprocessed prelamin A peptides (*lower panel, right lane*). **e** Reduction in the number of unrepairable DSB foci by pretreatment of HGPS cells with FTI (results of 6 Gy exposure and 1 week incubation). **f** HeLa S3 nuclei expressing GFP-progerin (*left panel*). DAPI staining of mock transfected HeLa nuclei (*right panel*)

Since it is reported that the repair of DSB foci (monitored by 53BP1 foci) is slower in dysmorphic nuclei in HGPS cells [28], we thought dysmorphic cells would also be abnormal with regard to the number of unrepaired foci. The results on the number of unrepaired 53BP1 or γH2AX foci per nucleus were sorted according to the ESR ratio of each nucleus. It was found that the number of unrepaired foci that persisted 1 month after exposure to 6 Gy under non-dividing conditions (6-Gy/1-Mo) was nearly twice as large in both HGPS and MJ90 cells (MJ is a normal strain) with dysmorphic nuclei (ESR ratio >1.05) when compared with that of cells with smooth nuclear contour (ESR ratio <1.015). The difference between MJ vs. HGPS cells was statistically significant only for the cells with dysmorphic nuclei (Figs. 2a, b).

It is reported that treatment of HGPS cells with inhibitors of protein farnesylation, farnesyl transferase inhibitor (FTI), ameliorates their abnormal nuclear shape through attenuating the progerin anchoring to nuclear membrane [22, 26, 29–31]. Indeed, following treatment of AG11513 cells (PDs 20) with FTI, the frequency of dysmorphic nuclei decreased to nearly 1/3 that of untreated control cells (Fig. 2c). We thus treated the HGPS cells with FTI for 3 days before irradiation and examined if the treatment led to any improvement in DSB repair. In the 6 Gy-irradiated and 1-week incubated cells (6-Gy/1-week), where basic bootstrap test resulted 2.6-fold increase of unrepaired foci in HGPS cells (95 % CI: 2.2–3.2), FTI treatment resulted in a significantly decreased number of unrepaired foci, and the results became closer to the level found in MJ cells (Fig. 2e, *p* < 0.01). The results clearly indicate that suppression of progerin accumulation onto nuclear membrane results in decreased number of unrepaired DSB foci in HGPS cells, and the response becomes closer to that of normal cells. Nuclear morphology and protein levels of lamin A/C and progerin following FTI treatment of HGPS cells are shown in Fig. 2d. It shows that FTI treatment improved nuclear morphology and the cells looked healthier. The drug also inhibited processing of normal lamin A, thus its precursor peptide is seen, along with the reduction of the progerin level. Also among non-irradiated HGPS cells at early passages, we noticed that there existed occasionally dysmorphic cells and they expressed high levels of progerin and already contained unrepaired R-DSB foci (Additional file 2: Figure S2 and Additional file 3: Figure S3).

Single-copy integration of GFP-progerin plasmid in HeLa S3 cells resulted in changes of their nuclear shape with lobulations (Fig. 2f) as already reported by [22, 32]. The result indicated that single copy-based, moderate expression of progerin still allowed the cells to grow, which was reminiscent of HGPS status.

### Ectopic over-expression of progerin completely inhibits an initial step of M-DSB repair

**Fig. 3:**
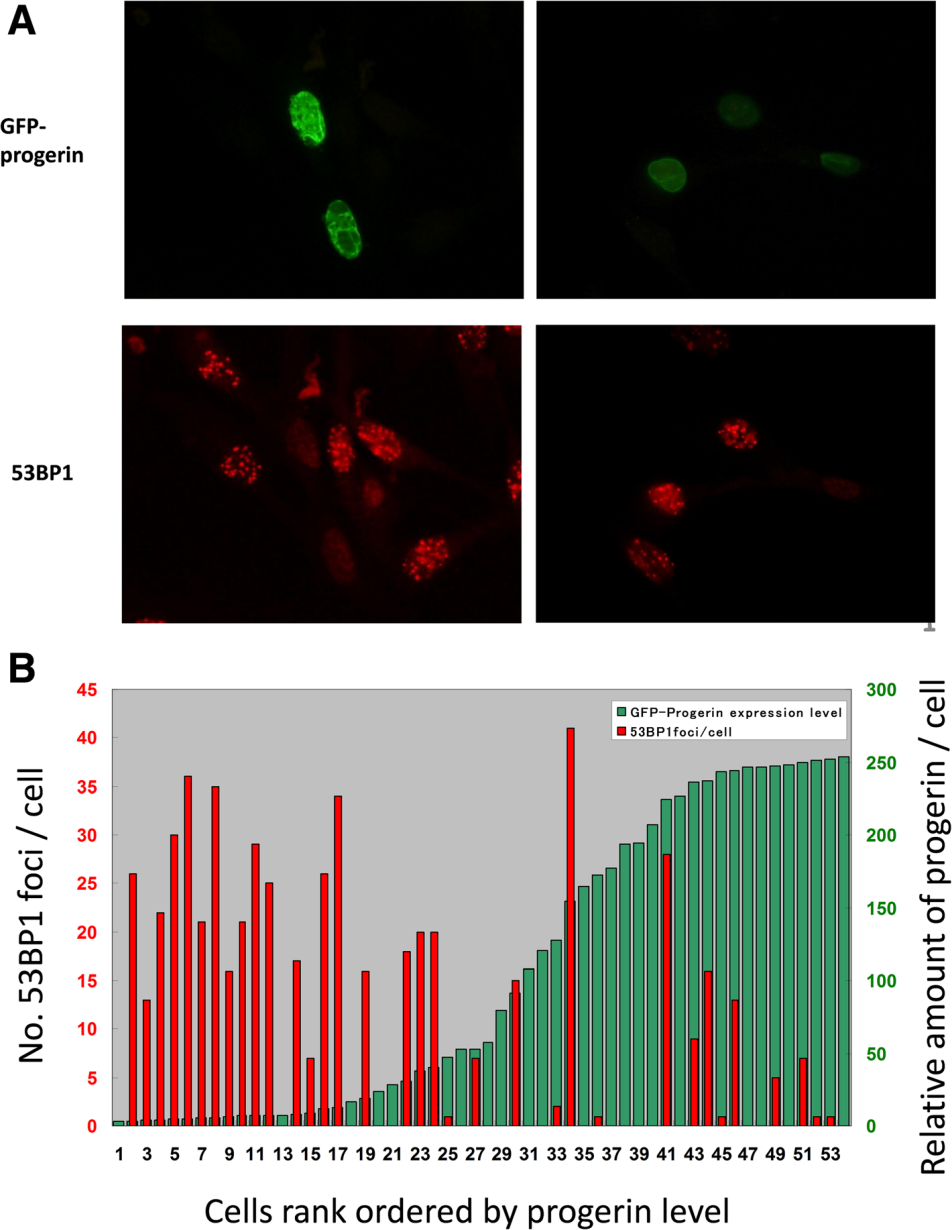
Inhibition of DSB repair foci formation by transient expression of GFP-progerin in NHDFs. **a** GFP-progerin plasmids were transiently expressed in early passage of MJ90 cells by lipofection, and formation of 53BP1 foci was monitored 1 h after exposure to 1 Gy (peak stage of repair foci formation). Typical examples of cell nuclei which showed strong (*left*) or weak (*right*) expression GFP-progerin are shown. **b** 53BP1 repair foci are predominantly formed in nuclei which expressed low levels of progerin (1 Gy exposure and 1 h incubation). Cells are rank-ordered by intensity of green fluorescence

To cause conditions of strong and forced expression of progerin, lipofection-mediated transient transfection method was applied to NHDF cells. In this experimental setting, over-expression of the exogenous gene is sustained through the introduction of multiple copy numbers of plasmids into the nucleoplasm. Following overnight transfection, NHDFs were subjected to 1 Gy irradiation, incubated for 1 h, and scored for number of repair foci. Interestingly, cells expressing high levels of GFP-progerin had no repair foci at all, whereas those which did not incorporate the plasmids formed a large number of 53BP1 foci (Fig. 3a, left panel). The contrast was so clear but appeared confusing because the HGPS cells exhibited normal DSB-repair kinetics after 1 Gy exposure (Fig 1a). Then, closer observation revealed that those cells which showed weak expression of GFP-progerin had normal levels of repair foci as already seen (Fig. 3a right panels). These observations clearly indicate that cells can normally repair radiation-induced DSBs, i.e. M-DSBs, when expression levels of progerin are moderate or low, but they reduce the DSB sensing capability when expression levels are excessive. Ectopic expression of GFP did not show such effect but GFP-lamin A exhibited to some extent (Additional file 4: Figure S4). As we observed a clear inverse relation between the expression level of progerin and the number of 53BP1 repair foci 1 h after exposure to 1 Gy (Fig. 3b), it is concluded that moderate expression of progerin represents the conditions of HGPS cells, and further expression of progerin is inhibitory to the formation of DSB repair foci, the first step toward repairing M-DSBs.

### Nuclear localization of progerin and unrepairable DSBs

**Fig. 4:**
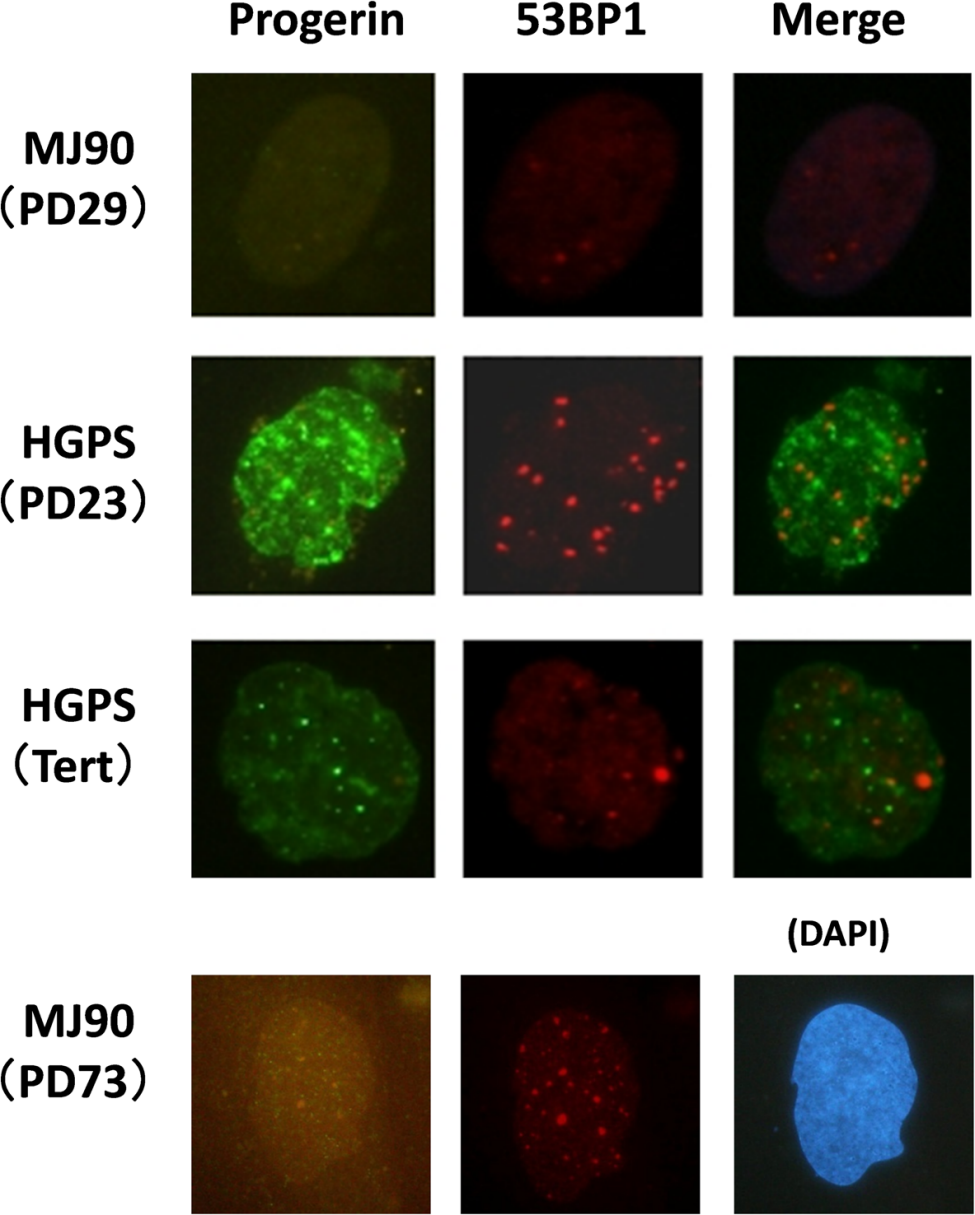
Nuclear localization of progerin and unrepairable 53BP1 foci. Progerin foci (aggregates) as detected by anti-progerin staining were superimposed onto typical 53BP1 foci. Note that the green color is not GFP, but antibody staining against progerin. Brightness of MJ90 (PD73) was enhanced to see the weak foci of progerin

Staining HGPS cells with an antibody specific to progerin (but which does not react to normal lamin A) identifies aggregates of progerin (or progerin foci) in the nuclei. This is more prominent in nuclei showing advanced dysmorphology. If it is assumed that progerin expression is the direct cause of increased levels in the number of unrepaired foci, it appears possible that unrepaired foci might co-localize with progerin foci. However, the results did not support this possibility (Fig. 4; HGPS PD23). The results may be explained by a high level of local accumulation of progerin inhibiting formation of 53BP1 repair foci, and thus no repair foci co-localized with progerin foci. Further study is required to address this issue.

### Telomerase induces cell rejuvenation

**Fig. 5:**
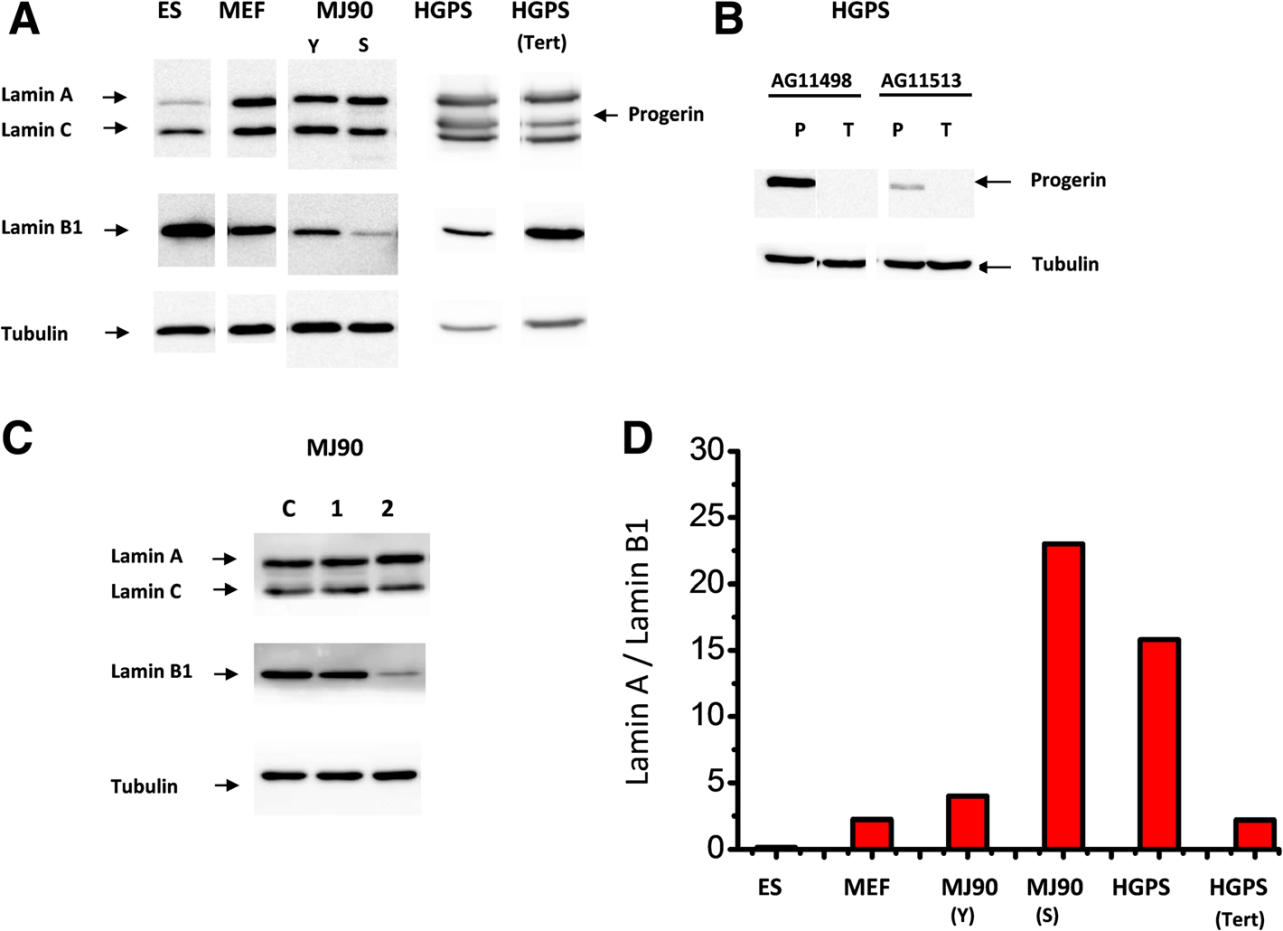
Cell aging and nuclear lamins. **a** Western analyses of lamin isoforms in different cells. ES, embryonic stem cells (ES-R1); MEF, mouse embryonic fibroblasts; Y (young) and S (senescent) MJ90 cells; HGPS cells (AG11513) and its immortalized line by hTERT, HGPS(Tert). **b** Western analysis of progerin. P, parental; T, hTERT immortalized cells. **c** Irradiation-associated aging causes decreased expression of lamin B1 in early passages of MJ90 cells. Lane C represents untreated cells, and lanes 1 and 2 represent 12 Gy-irradiated cells with post-irradiation incubation of 1 day or 3 months, respectively. **d** Ratio of lamin A/lamin B1 in cells at various conditions; see legend to Fig. 5a

**Fig. 6:**
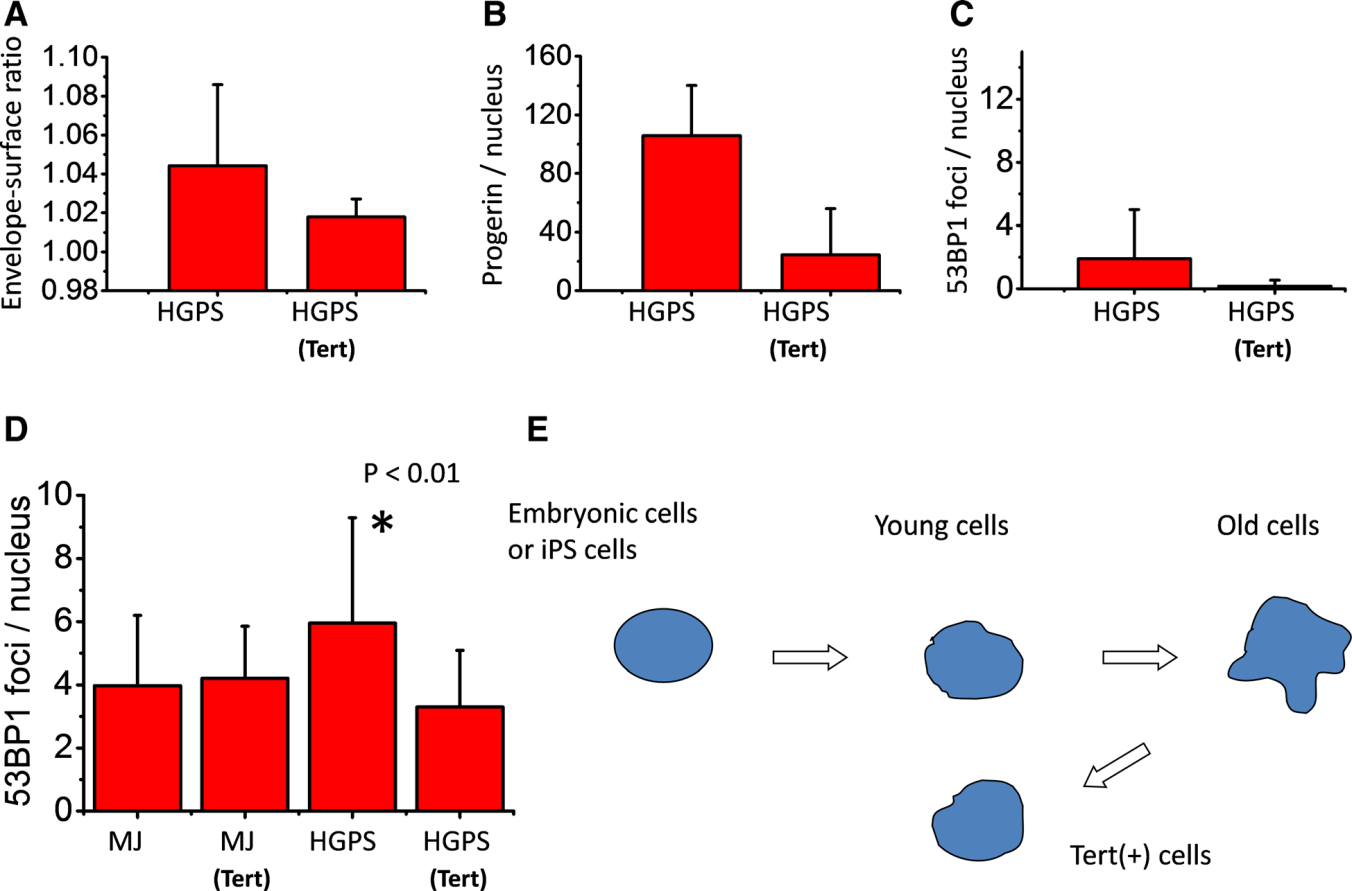
Rejuvenation of HGPS cells by enforced expression of human telomerase gene (hTERT). **a** Envelope-surface ratio (ESR) of nuclei. **b** Expression level of progerin. **c** Number of 53BP1 foci per nucleus in HGPS cells (AG11513 cells at PDs ~23) and the hTERT-introduced cells (PDs ~110). **d** Number of unrepairable repair foci at 12-Gy/ 1-month. **e** Proposed model of telomerase mediated rejuvenation

Since the replicative life span of HGPS cells is very short and one of the major causes of accelerated senescence of HGPS cells is thought to be attributable to earlier telomere attrition [33, 34], we wanted to extend their in vitro life span with the introduction of telomerase (hTERT). As a result, the two HGPS cell strains were successfully immortalized as they had attained more than two years of continuous culture, and the number of population doublings exceeded ~200 (by far beyond their ordinal life span of 20 to 40 PDs). It seems difficult to imagine that addition of telomere sequences could be the sole cause of this success without circumventing adverse conditions in the nuclei. Indeed, the immortalized HGPS cells with hTERT exhibited various changes, such as remarkable improvement of nuclear shape, down-regulation of progerin expression, and decreased number of persisting 53BP1 foci under steady-state conditions (Fig. 4; HGPS Tert, Fig. 5b, Fig. 6a, b, c). The results indicate that telomerase-mediated immortalization requires improvement of aging phenotypes, or the improved phenotypes are the results of reversal from the aging process. In the normal processes of aging, senescent NHDFs (PDs ~73) produce progerin, but at a very low level [Fig. 4, MJ90 PD73 (Note that enlarged view of MJ90 PD73 shows existence of progerin foci.); Fig. 5)]. However, the frequency of dysmorphic nuclei progressively increased as the normal cells reached senescence (Fig. 4; MJ90 PDs 73, and Additional file 1: Figure S1). In contrast, levels of progerin mRNA determined by real time RT-PCR did not show apparent differences between young and senescent NHDFs (Additional file 5: Figure S5A). Conflicting results are reported on this issue [14, 35].

Generation of iPS cells from HGPS fibroblasts resulted in healthy undifferentiated embryonic cells with normal nuclear shape [36, 37]. This was possible because the induced undifferentiated cells did not express lamin A but instead lamin B1 supported the nuclear membrane meshwork. Lamin B1 is another structural component of nuclear lamina in somatic stem cells and embryonic cells, and the lack of lamin A expression means no production of progerin [36]. Switching iPS cells toward differentiation starts induction of lamin A, and so does the production of progerin, which leads to accelerated aging in the HGPS-iPS cells. Therefore, we expected enhanced lamin A expression to occur also during aging processes of normal cells along with an increased expression of progerin. Contrary to expectation, the results showed decreased expression of lamin B1 (Fig. 5a). That trend is true for radiation-induced premature-senescent NHDFs (Fig. 5c; down regulation of lamin B1 in 12-Gy/3-month cells). As shown in Fig.5a and d, relative amount of lamin B1 expression was already down-regulated in early passages of HGPS cells. Our result indicates that cellular aging is expressed as a process of increasing the ratio of lamin A/lamin B1 (Fig. 5d). If so, what can be the role of telomerase? Following immortalization of HGPS cells with hTERT, the progerin level decreased extensively (Fig. 4, 5b, and 6b). Moreover, in the progerin specific Western analysis, it decreased to 1/100 that of the parental cells (Fig. 5b). Accordingly, both the frequency of dysmorphic nuclei and the number of 53BP1 foci decreased (Fig. 6a, b, c). Production of radiation-induced unrepairable DSBs were also reduced (Fig. 6d).

Therefore it is now clear that telomerase is not simply an enzyme that adds telomere repeats at chromosome ends but also it has an important role in cell reversal from senescence. The lamin A/ lamin B1 ratio of HGPS(Tert) cells was found to have returned to the level of young, normal fibroblasts (Fig. 5d). It is possible that a model proposed by Scaffidi and Misteli [14] may be correct; namely, normal processes of aging consist of abnormal accumulation of lamin A to nuclear membrane, which leads to an increased abnormality of nuclear shape. In fact, lamin A tends to accumulate in nuclear membrane in aged normal cells (Additional file 6: Figure S6). They indicated that this is caused by the small amount of progerin, but further studies are required to substantiate the hypothesis.

## Discussion

The major findings of the present study are: 1) HGPS cells are able to repair radiation-induced M-DSBs as efficiently as normal cells when examined shortly after irradiation (e.g., 1 day), whereas the number of residual, unrepaired foci (R-DSBs) in the cells is greater than that in normal cells after a long post-irradiation incubation (e.g., 2 weeks to months). This could be contrasted with previous reports demonstrating slower repair of DSBs in HGPS cells [24, 28]. Possible reasons for this inconsistency in reports may be differences in cell strains used and/or in radiation doses used ([24, 28], 5 Gy compared to our 1 Gy). However, even though the higher dose produces much more DSBs, the initial step of the damage recognition would not be changed. In this context, another possibility is that the HGPS cells they used had more advanced stages of nuclear dysmorphology. We also noted that even in unirradiated young HGPS cultures, the fraction of cells bearing unrepairable foci were readily seen (Additional file 2: Figure S2 and Additional file 3: Figure S3). These results are compatible with previous observations that cells expressing GFP-progerin gradually accumulate γH2AX foci [38]. 2) Cells expressing an excess amount of progerin cannot form M-DSB foci, which are generally formed within one hr after irradiation. With regard to the latter, our results are in line with those reported by Manju et al. [32], that an excess expression of progerin suppressed formation of γH2AX foci following cisplatin or ultraviolet (UV) treatment. Since repair enzymes involved in radiation-induced damage and chemical- or UV-induced damage are quite different, it is unlikely that progerin acts through suppression of specific repair enzyme(s) to affect the formation of repair foci. Rather, it is more likely that progerin acts primarily to affect the nuclear membrane and consequently disable the DNA-repair machinery associated with nuclear membrane. However, little is known about the connection between the nuclear membrane and the DNA repair system. A possible link comes from a recent report demonstrating impaired nuclear transport of macromolecules in HGPS cells and accumulation of progerin on the nuclear membrane. Progerin, through nuclear membrane dysfunction, inhibits RanGTPase activity resulting in down-regulation of Tpr, a loading cargo of nuclear pore. Thus transport of high molecular weight proteins into the nucleus is impaired [39, 40]. In this context, core proteins of DSB repair with large molecular size, such as DNA-PKcs (Mw 460 kD), ATM (350 kD) or ATR (300kD) might be inefficiently transported after the initial genotoxic insult. If this is the case, higher doses or repeated irradiation might render the cells unable to repair damage and produce enhanced levels of unrepaired DSBs. Indeed, trapping of DNA-PK by progerin has been described [36]. We also noted that ser-2056 phosphorylated forms of DNA-PK predominantly localized in nuclear membrane in HGPS cells after the irradiation (Additional file 7: Figure S7). However, the effect of membrane trapping of DNA-PK appeared marginal, since the cell clones stably expressing progerin did not exhibit elevated radiosensitivity (Additional file 5: Figure S5B).

Lamin A has multiple roles in the nuclear membrane as well as the nucleoplasm through interaction with many proteins [15, 29, 41–43]. It should also be noted that mouse embryonic fibroblasts (MEF) carrying a homozygous deletion of the lamin A gene also exhibited defects in the formation of M-DSB foci after X-ray irradiation [44]. These results strongly indicate that the level of lamin A has to be carefully balanced for DSB repair. Thus excess lamin A, or its forms of progerin, in the nuclear membrane has deleterious consequences similar to the effects of low levels of the protein. We noted, as previously reported by others, [34, 38] that over-expression of a wild-type lamin A-GFP construct also induced some nuclear deformation (Additional file 8: Figure S8).

Introduction of the hTERT gene into HGPS cells induced immortalization, with accompanying changes such as smoother nuclear shape and decreased number of 53BP1 foci, under steady-state conditions. This could be due to the fact that cells bearing unrepairable DSBs were incapable of undergoing cell division following telomerase expression, and were excluded from the culture during the immortalization process (positive selection of relatively young cells). Alternatively, telomerase may protect the HGPS cells from the spontaneous formation of unrepairable DSBs, or the protein may change cells to be able to manage to repair, overcome, or adapt to the unrepaired DSBs for the sake of continuous growth.

Since the R-DSBs might be physicochemically distinct from the M-DSBs, they might be distinctly localized in specific sites in the nuclear architecture. In yeast, DSBs difficult to repair are recruited to the nuclear periphery for processing by a special repair complex [45, 46] which might include nuclear lamins, the nuclear pore protein complex, and telomere protein components [15, 42, 43, 47, 48]. Indeed, in our study, FTI treatment resulted in improved nuclear shape associated with decreased numbers of unrepaired DSBs in irradiated HGPS cells (Fig. 2), even though the target of the drug cannot be confined to membrane-bound lamin A. Our observations may indicate that localized sites of nuclear membrane (or a possibility of heterochromatin attached to the membrane) involve DSB repair sites, or conversely unrepaired DSBs may induce altered nuclear architecture to facilitate nuclear bending or lobulation. Further studies are required to clarify the issue.

During embryonic development, lamins are closely related to the molecular switches of cell differentiation. In undifferentiated stem cells, nuclear membranes are structurally supported by lamin B1, but not lamin A. Thus, undifferentiated somatic cells of HGPS patients are normal in physiologic functions. Upon triggering of a signal for differentiation, cells start transcribing the lamin A gene, including mutant progerin transcripts, and exhibit accelerated aging. Consistent with this is the fact that Lamin A knockout mice are runt and can only survive until soon after weaning [49], a time at which many types of somatic cells begin differentiating to produce functional tissues. This has led to the suggestion that the aging switch is under the control of expression of the lamin A gene [36, 50]. However, our results show that an age-related decrease in the expression of lamin B1 was more pronounced in HGPS versus normal cells indicating that changes in the ratio of lamin A/lamin B1 (Fig. 5) are involved in the progression to cell senescence. This is further supported by recent studies, which demonstrated that lamin B1 gene silencing induced premature senescence that was accompanied by a decrease in cell proliferation, whereas the gene activation induced cell growth [51, 52]. We have found that normal cells which undergo radiation-induced premature senescence show a decreased expression of lamin B1 whereas expression levels of lamin A and C are unchanged (Fig. 5c, lane 2). This result is similar to the observations made following natural aging of normal cells (Fig. 5a, MJ90 lane S). Our hypothesis that lamin A/lamin B1 ratio can be regarded as an index of cellular aging is the same as proposed by Freund and others [53]. We also noted that both the HGPS cells and radiation-induced senescent cells up-regulated p21 but not Chk2. (Additional file 9: Figure S9). This condition is similar to normal aging (MJ senescent in Additional file 9: Figure S9), as it is already reported [54].

## Conclusions

In conclusion, we have verified our hypothesis that lamin A combined with the progerin-associated nuclear envelop strongly controls cell aging associated with DNA damage.　The present study also reveals that some radiation-induced DNA damage could be related to a common mechanism underlying natural aging, and that the exaggerated aging phenotypes of HGPS are, in part, caused by a more rapid accumulation of such DNA damage, or by more frequent emergence of cells bearing such damages because of imperfect DNA repair. Furthermore, the maintenance of cell hierarchy against aging is achieved through removal of such unrepairable damage.

## Additional files

Additional file 1: Figure S1. Nuclear shapes of senescent NHDFs (MJ90 cells at PD 73) with DAPI staining. (TIFF 8387 kb) Additional file 2: Figure S2. HGPS cell population at PD14 already contains cells with high expression of progerin (green) and 53BP1 foci (red). (TIFF 6466 kb) Additional file 3: Figure S3. Different expressions of 53BP1 foci (left) or progerin (right) in relation to nuclear dysmorphology. Individual HGPS cells (AG11513 at PD14) were assessed for the envelope-surface ratio (ESR), the number of 53BP1 foci, and progerin expression. (TIFF 1555 kb) Additional file 4: Figure S4. Supplement to Fig. 3a. Expression of GFP (top panel) or lamin A-GFP (middle panel) does not inhibit formation of 53BP foci following 1 Gy irradiation but expression of progerin-GFP does. (TIFF 5121 kb) Additional file 5: Figure S5. (A) Measurement of progerin mRNA levels with real-time RT-PCR. Progerin-specific PCR primers (gtgaggaggacgcaggaa, gacgcaggaagcctccac, 87 bp product, Scaffidi & Misteli, Science 312:1059–1063, 2006), and lamin A-specific primers (ggtggtgacgatctgggct, ccagtggagttgatgagagc, 126 bp product) were used along with primers specific to human XPA gene (agcagtaagaagcagcgtgt, acaagtcttacggtacatgt) as the internal control. Relative levels of progerin messages were corrected by the expression level of lamin A. (B) X-ray dose survival curves of various cell clones bearing a single copy of the GFP-progerin gene. HeLa S3 stands for the non-transfected parental cells. (TIFF 1615 kb) Additional file 6: Figure S6. A cross-sectional view of lamin A localization indicates its association with nuclear membrane in both young and senescent NHDFs. (TIFF 3823 kb) Additional file 7: Figure S7. Phosphorylated form of DNA-PK was predominantly localized in nuclear membrane fraction in HGPS cells after irradiation. (TIFF 2104 kb) Additional file 8: Figure S8. Over-expression of wild-type lamin A also induced nuclear deformation to some extent in HeLa cells. Note that wild-type lamin A over-expression could not result in clear nuclear edge formation. This is a remarkable difference from that of progerin over-expression. (TIFF 13258 kb) Additional file 9: Figure S9. Cell cycle status of senescent cells. Both replicative and radiation-induced senescent cells produced enhanced level of p21 but phosphorylated form of Check 2 was absent. (TIFF 2062 kb)
