# Supplementary figures and images for: Progerin, the protein responsible for the Hutchinson-Gilford progeria syndrome, increases the unrepaired DNA damages following exposure to ionizing radiation
Source: Genes Environ. 2015 Oct 1;37:13. doi: 10.1186/s41021-015-0018-4 (PMC4917958; doi:10.1186/s41021-015-0018-4)

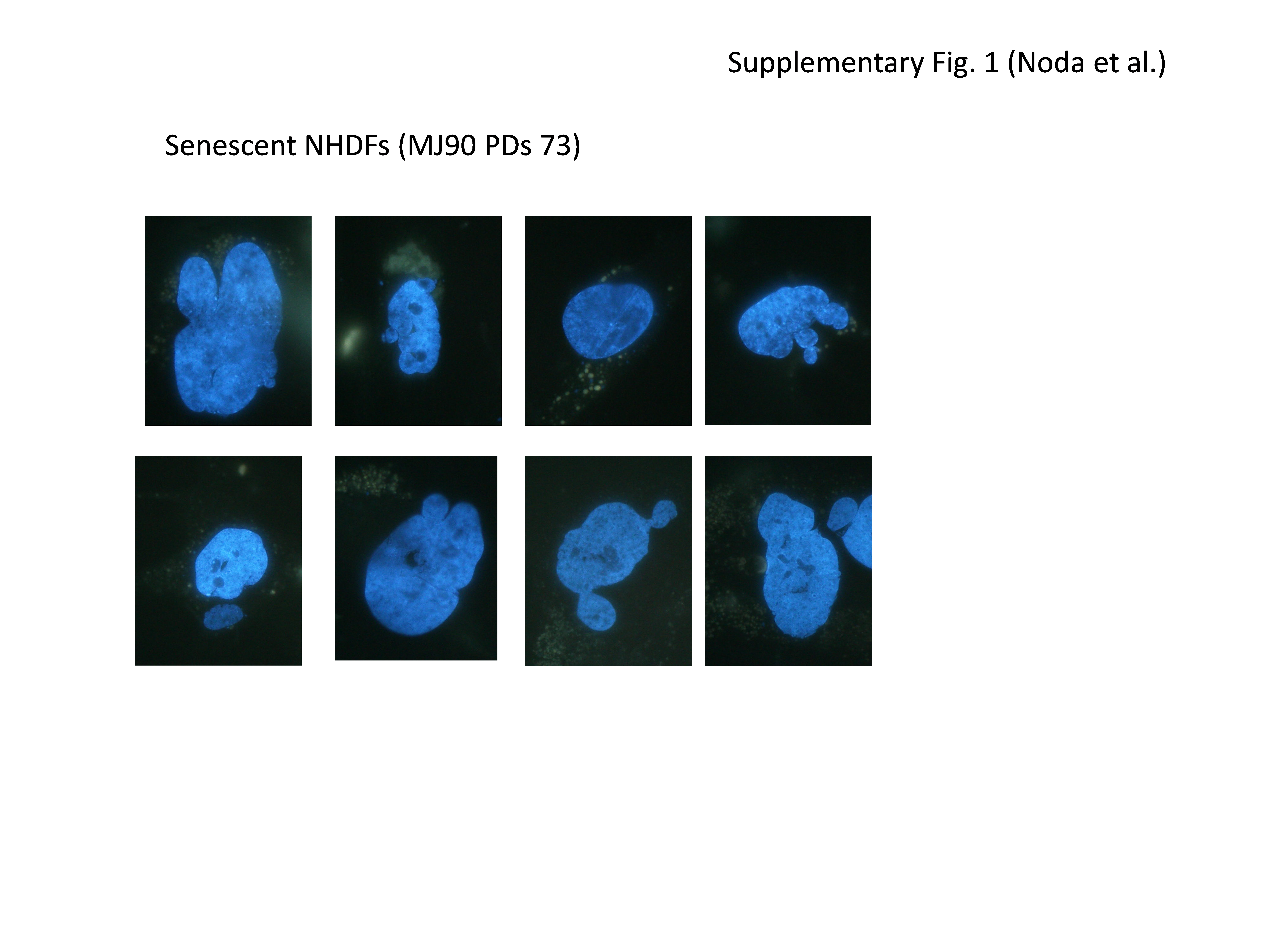

Supplement: Additional file 1: Figure S1. — Nuclear shapes of senescent NHDFs (MJ90 cells at PD 73) with DAPI staining. (TIFF 8387 kb) [file 41021_2015_18_MOESM1_ESM.tif]

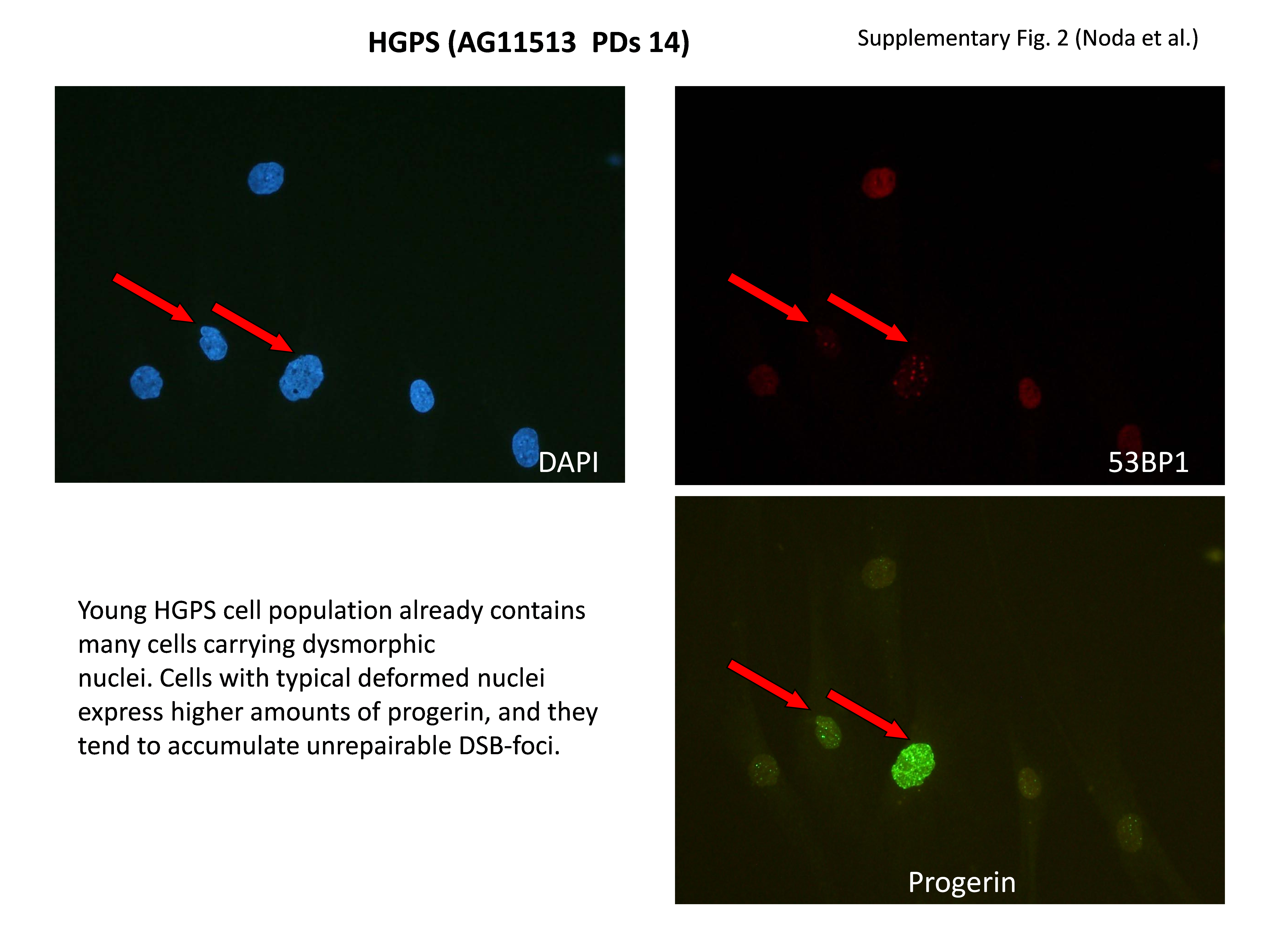

Supplement: Additional file 2: Figure S2. — HGPS cell population at PD14 already contains cells with high expression of progerin (green) and 53BP1 foci (red). (TIFF 6466 kb) [file 41021_2015_18_MOESM2_ESM.tif]

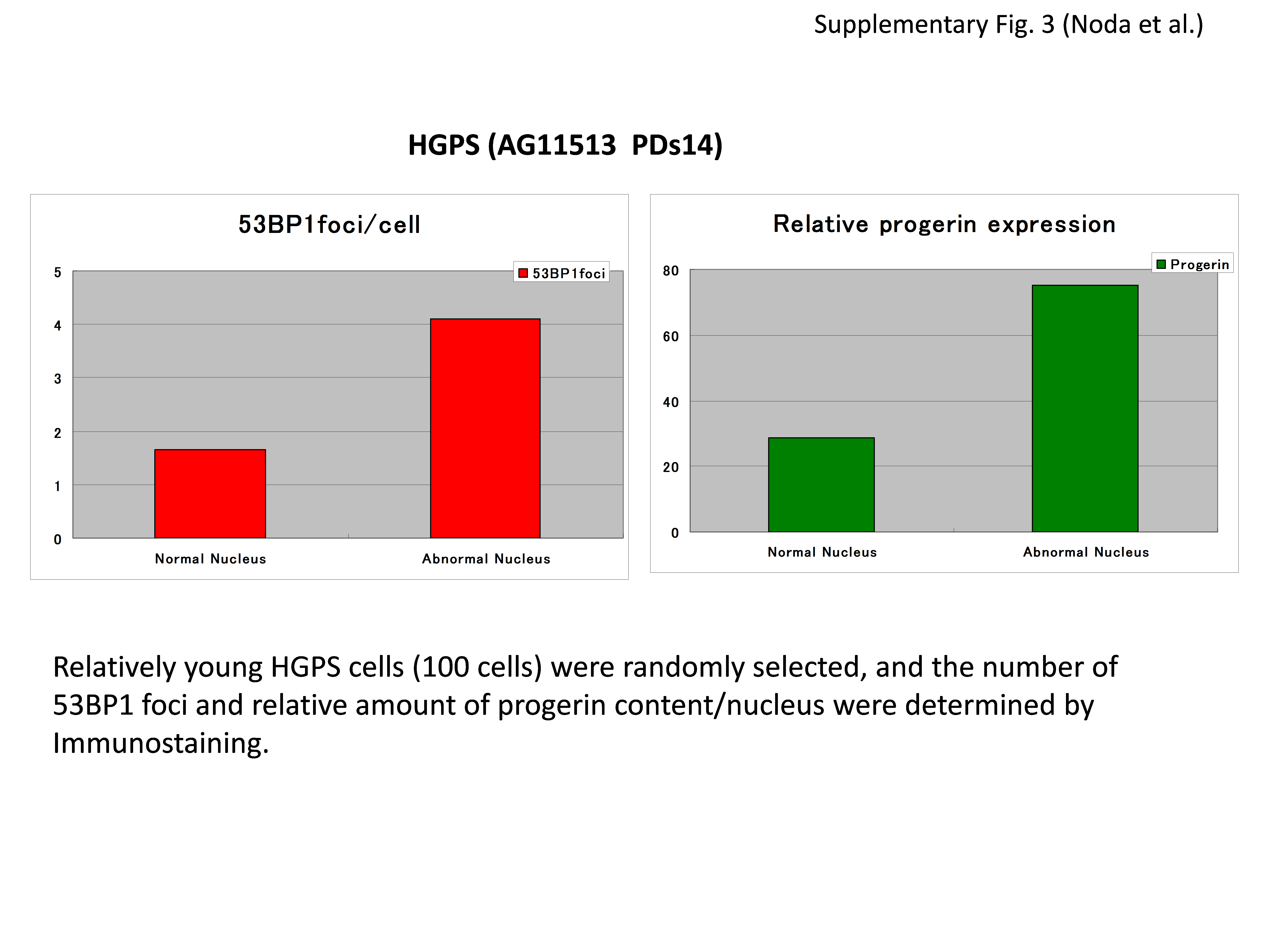

Supplement: Additional file 3: Figure S3. — Different expressions of 53BP1 foci (left) or progerin (right) in relation to nuclear dysmorphology. Individual HGPS cells (AG11513 at PD14) were assessed for the envelope-surface ratio (ESR), the number of 53BP1 foci, and progerin expression. (TIFF 1555 kb) [file 41021_2015_18_MOESM3_ESM.tif]

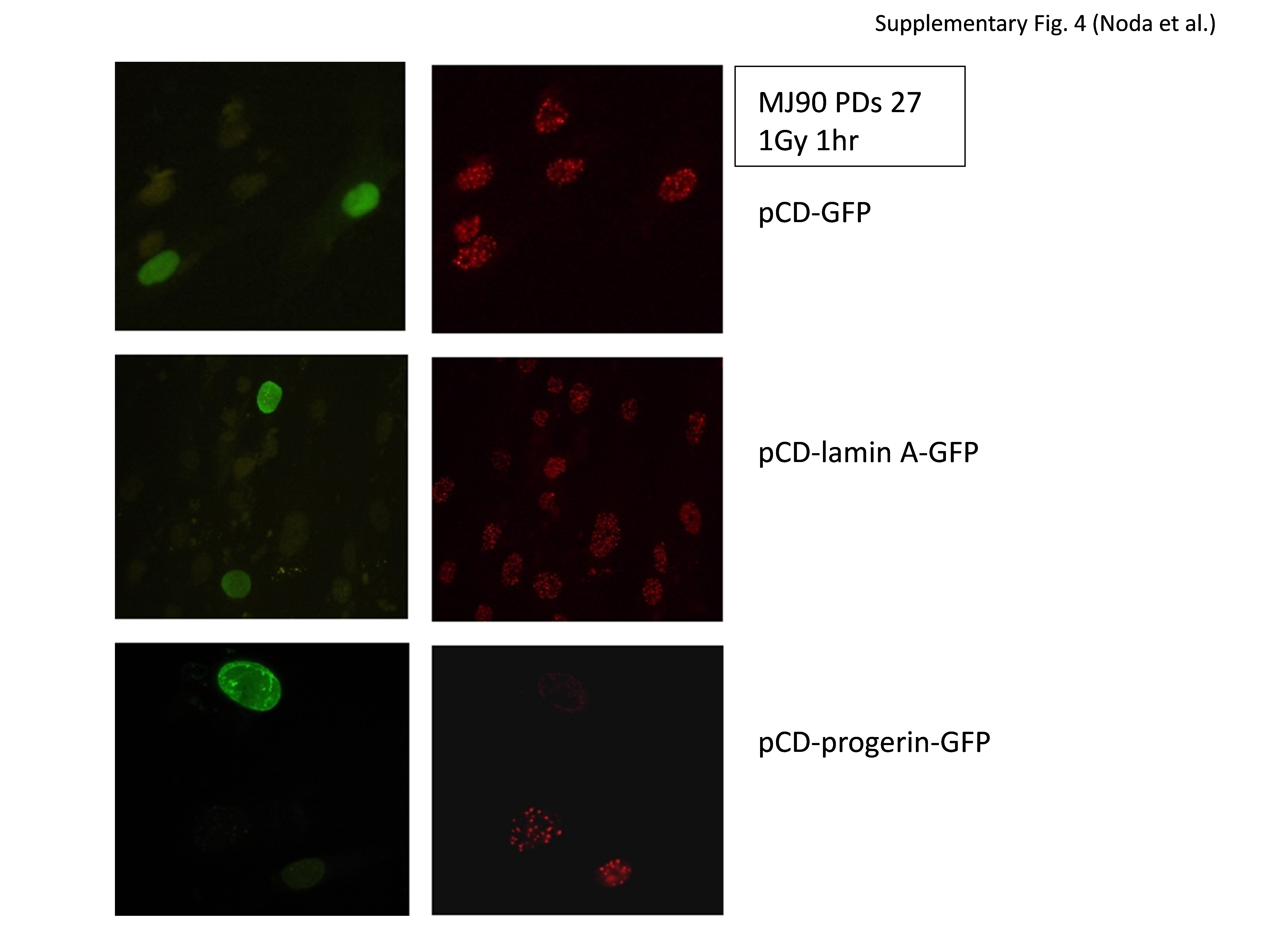

Supplement: Additional file 4: Figure S4. — Supplement to Fig. 3a. Expression of GFP (top panel) or lamin A-GFP (middle panel) does not inhibit formation of 53BP foci following 1 Gy irradiation but expression of progerin-GFP does. (TIFF 5121 kb) [file 41021_2015_18_MOESM4_ESM.tif]

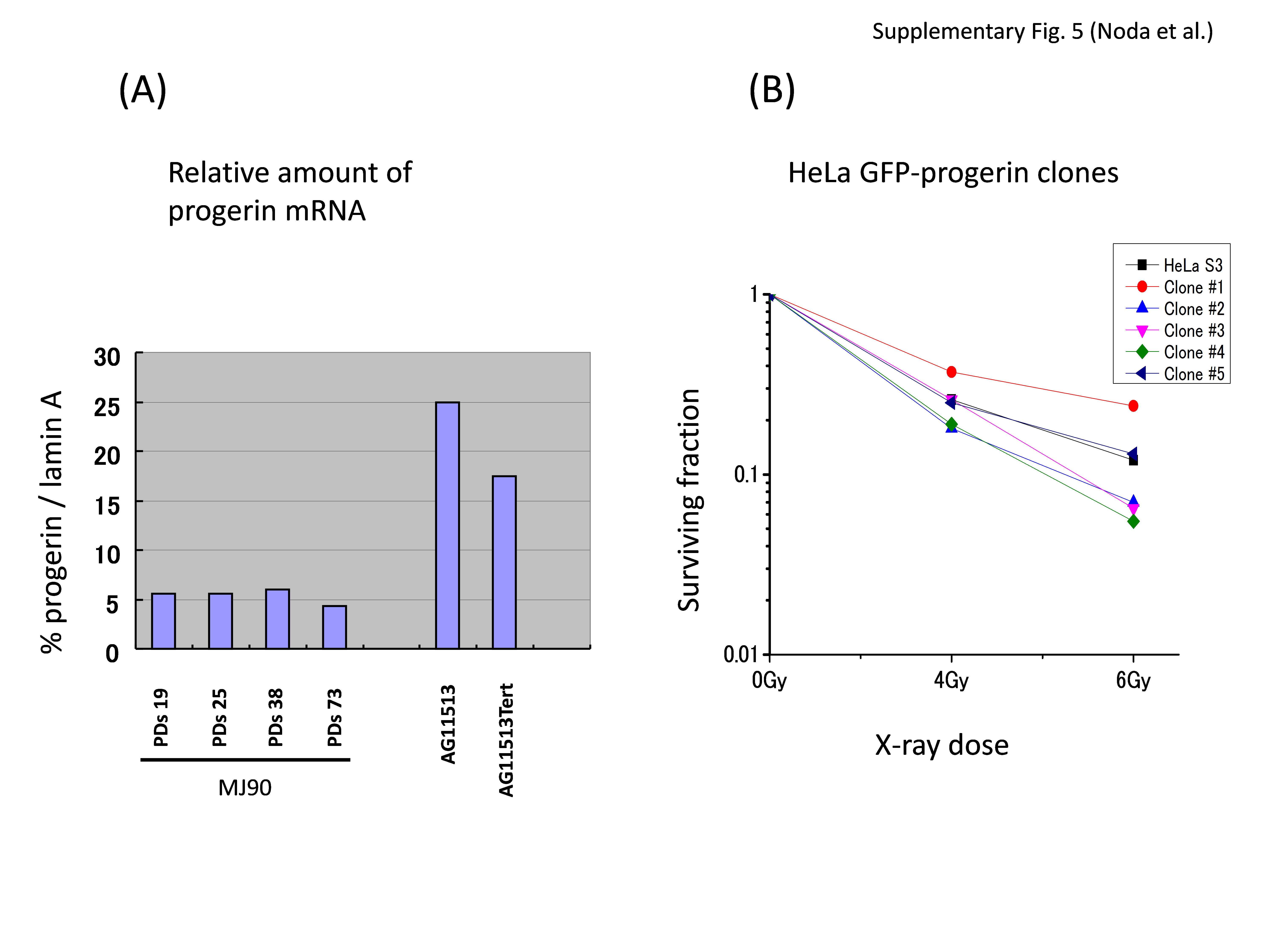

Supplement: Additional file 5: Figure S5. — (A) Measurement of progerin mRNA levels with real-time RT-PCR. Progerin-specific PCR primers (gtgaggaggacgcaggaa, gacgcaggaagcctccac, 87 bp product, Scaffidi & Misteli, Science 312:1059–1063, 2006), and lamin A-specific primers (ggtggtgacgatctgggct, ccagtggagttgatgagagc, 126 bp product) were used along with primers specific to human XPA gene (agcagtaagaagcagcgtgt, acaagtcttacggtacatgt) as the internal control. Relative levels of progerin messages were corrected by the expression level of lamin A. (B) X-ray dose survival curves of various cell clones bearing a single copy of the GFP-progerin gene. HeLa S3 stands for the non-transfected parental cells. (TIFF 1615 kb) [file 41021_2015_18_MOESM5_ESM.tif]

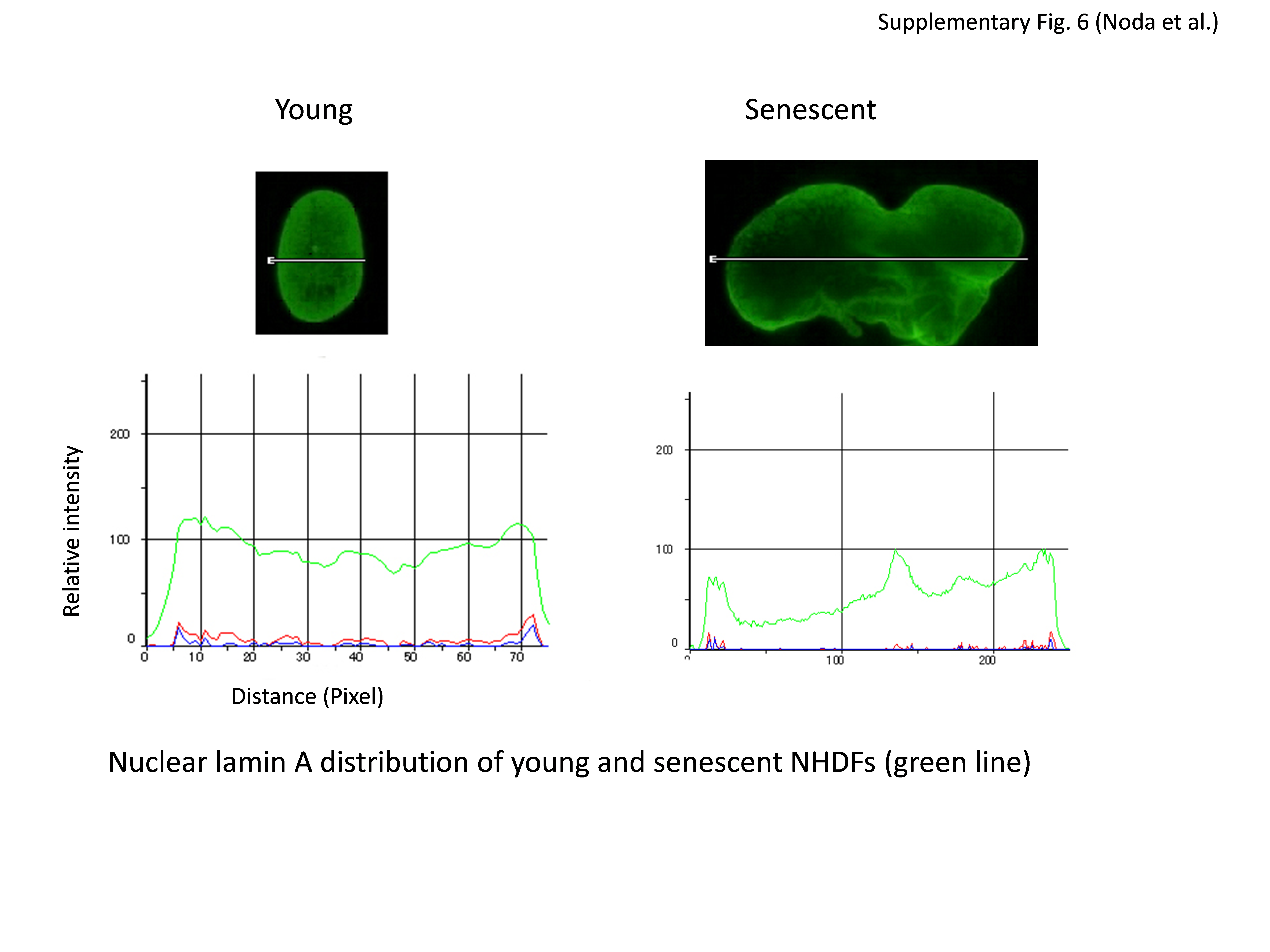

Supplement: Additional file 6: Figure S6. — A cross-sectional view of lamin A localization indicates its association with nuclear membrane in both young and senescent NHDFs. (TIFF 3823 kb) [file 41021_2015_18_MOESM6_ESM.tif]

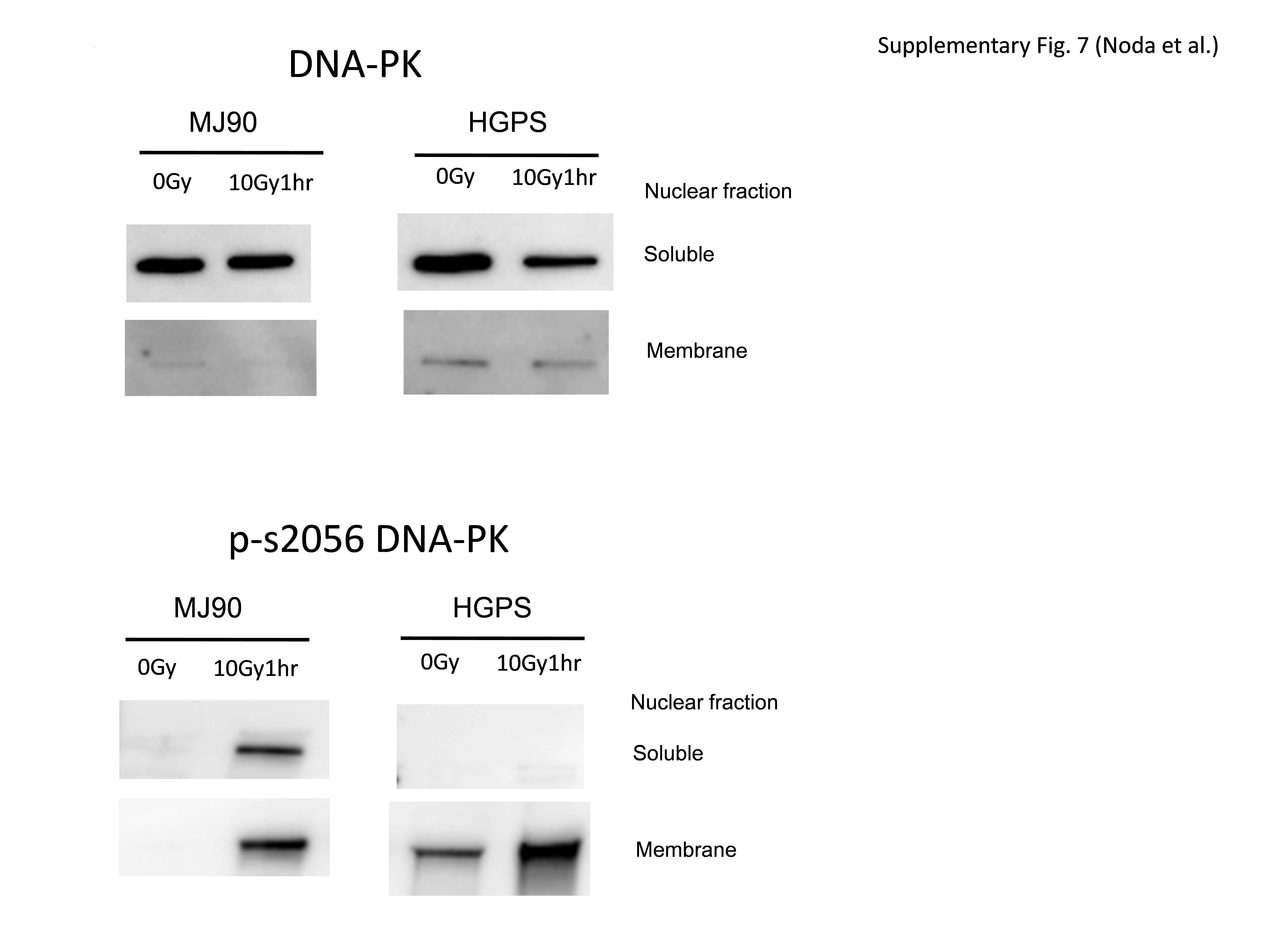

Supplement: Additional file 7: Figure S7. — Phosphorylated form of DNA-PK was predominantly localized in nuclear membrane fraction in HGPS cells after irradiation. (TIFF 2104 kb) [file 41021_2015_18_MOESM7_ESM.tif]

## Slide 1
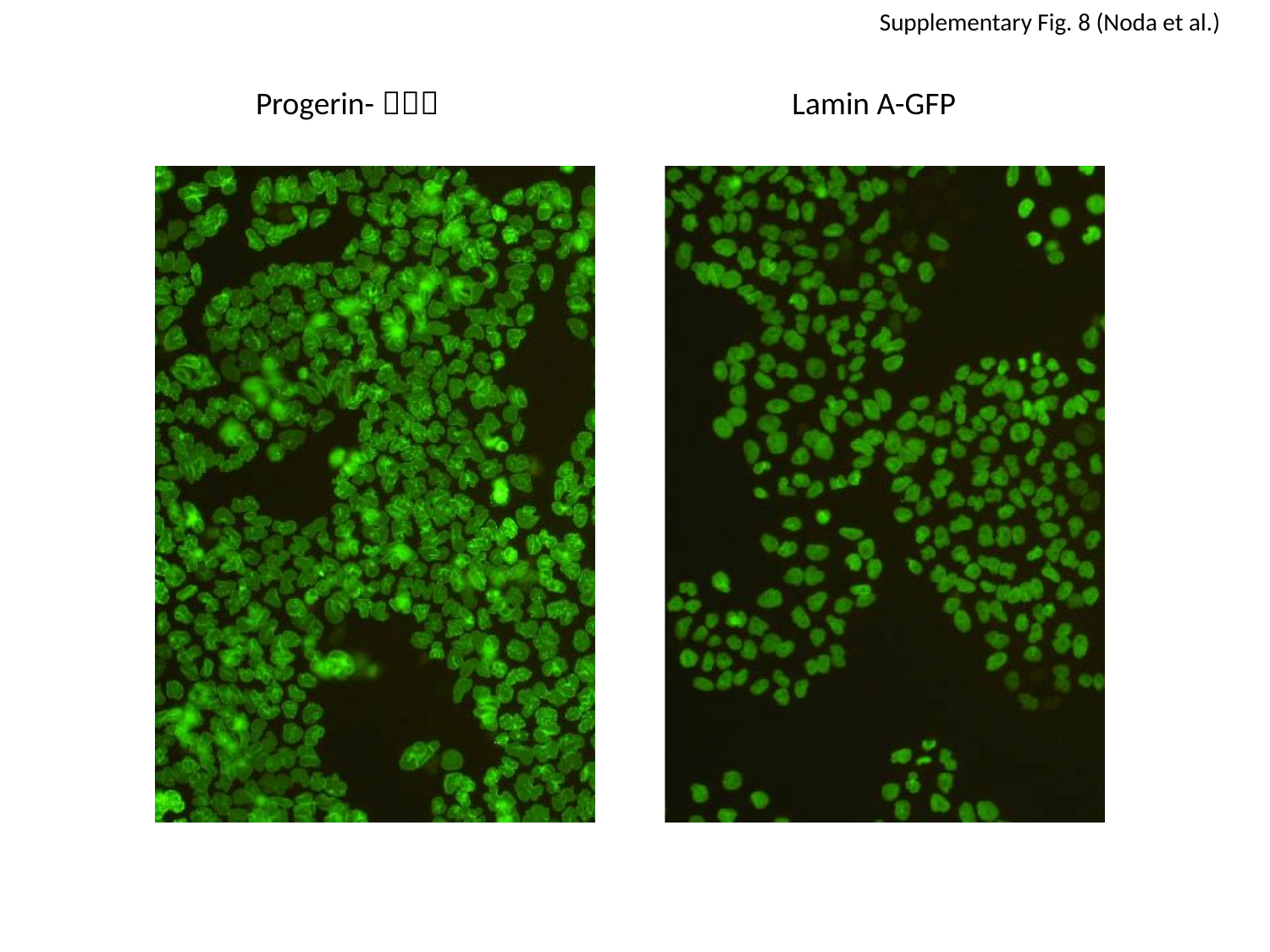

Supplementary Fig. 8 (Noda et al.)
Progerin-ＧＦＰ
Lamin A-GFP

Supplement: Additional file 8: Figure S8. — Over-expression of wild-type lamin A also induced nuclear deformation to some extent in HeLa cells. Note that wild-type lamin A over-expression could not result in clear nuclear edge formation. This is a remarkable difference from that of progerin over-expression. (TIFF 13258 kb) [file 41021_2015_18_MOESM8_ESM.pptx]

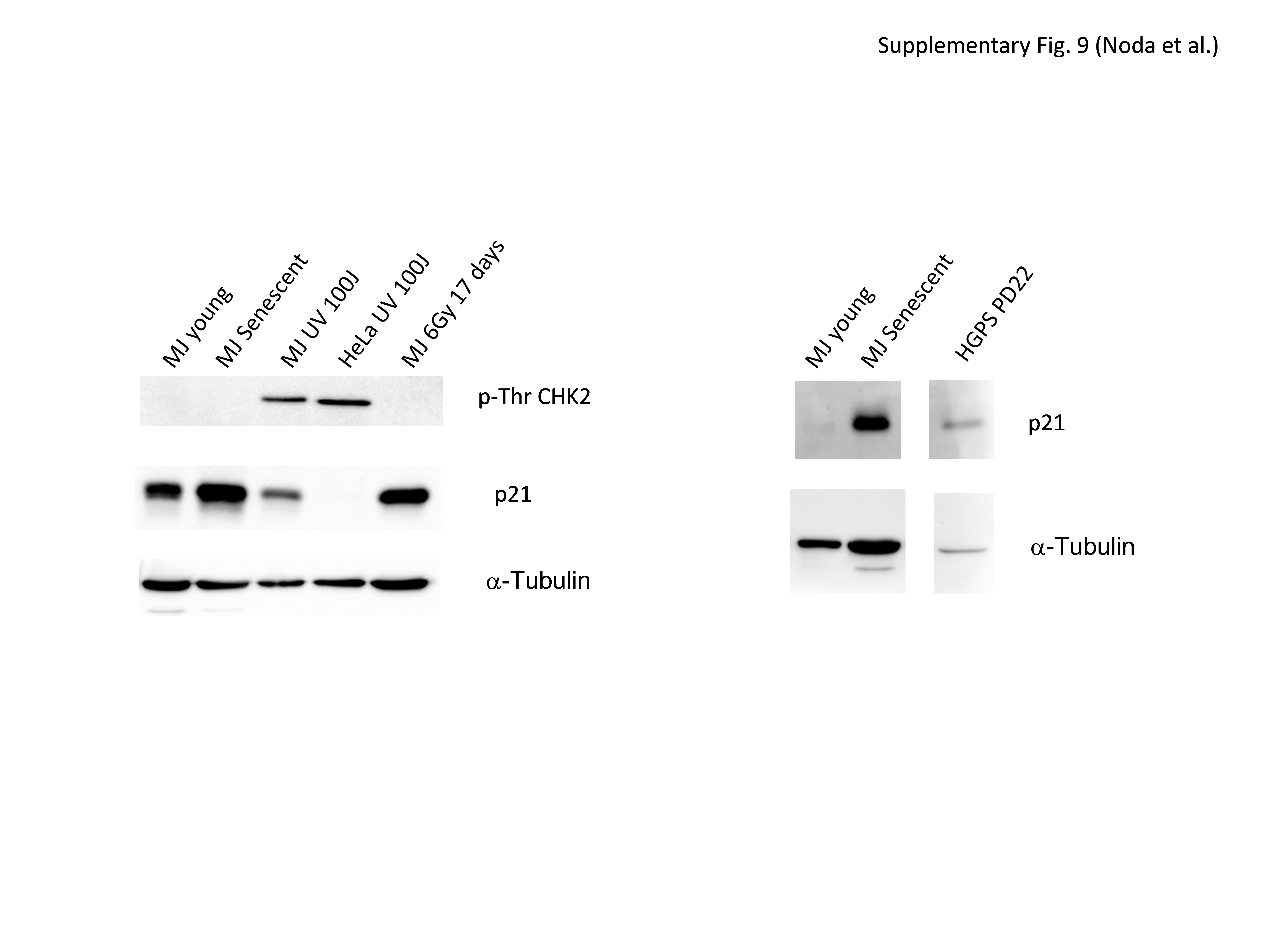

Supplement: Additional file 9: Figure S9. — Cell cycle status of senescent cells. Both replicative and radiation-induced senescent cells produced enhanced level of p21 but phosphorylated form of Check 2 was absent. (TIFF 2062 kb) [file 41021_2015_18_MOESM9_ESM.tif]
